# Intrathoracic Rhabdoid Tumor: A Rare Site in a Jordanian Infant

**DOI:** 10.7759/cureus.80520

**Published:** 2025-03-13

**Authors:** Maher Khader, Ruba Alhazaimeh, Mais Jazazi, Sura Alrawabdeh, Ayat Alalwan, Yara Alkafawin, Ayman Alhwayan, Waseem Almefleh, Hanadi Alkhalaileh, Haneen Alrawashdeh

**Affiliations:** 1 Department of Paediatric Haematology and Oncology, Royal Medical Services, Queen Rania Children's Hospital, Amman, JOR; 2 Department of Pathology and Laboratory Medicine, Royal Medical Services, Princess Iman Center for Research and Laboratory, Amman, JOR; 3 Department of Pediatric Radiology, Royal Medical Services, Queen Rania Children's Hospital, Amman, JOR; 4 Department of Pediatric Surgery, Royal Medical Services, Queen Rania Children's Hospital, Amman, JOR

**Keywords:** extrarenal extracranial rhabdoid tumor, intrathoracic neoplasm, malignant rhabdoid tumor, pediatric malignancies, smarcb1-deficient tumor

## Abstract

Intrathoracic rhabdoid tumors in infants are rare and aggressive malignancies that pose significant diagnostic and therapeutic challenges. We report the case of a previously healthy six-month-old full-term infant admitted with severe respiratory distress and desaturation, requiring admission to the pediatric intensive care unit (PICU) and intubation. Initial evaluation suggested left upper lobe pneumonia, but the patient demonstrated minimal improvement with antibiotics and corticosteroids. Then a chest CT was done and revealed a large, heterogeneously enhancing pleural-based mass compressing the left lung, initially interpreted as an aggressive pleural tumor, such as bronchopleural blastoma. The findings led to the urgent initiation of chemotherapy, resulting in temporary clinical improvement that allowed for extubation and a biopsy to be taken. The initial biopsy indicated a germ cell tumor with yolk sac based on these immunohistochemical markers: SALL-4, cytokeratin, and vimentin. However, the patient's lack of a sustained response to chemotherapy and subsequent clinical deterioration prompted a repeat biopsy, which confirmed the diagnosis of an intrathoracic rhabdoid tumor. Despite aggressive multimodal therapy, the patient developed liver metastases five months after the initial diagnosis and was transitioned to palliative care. This case highlights the tumor's complex behavior and resistance to therapy, and underscores the diagnostic challenges associated with intrathoracic masses in infants, particularly the overlap between germ cell tumors and rhabdoid tumors.

## Introduction

Malignant rhabdoid tumors (MRTs) are rare and highly aggressive neoplasms that predominantly affect infants and young children. First identified in 1978 as a sarcomatous variant of Wilms' tumor, MRTs were later classified as a distinct pathological entity due to their unique features [1]. These tumors are defined by their characteristic rhabdoid appearance under the microscope and the loss of the tumor suppressor gene SMARCB1/INI1, which plays a crucial role in chromatin remodeling and gene expression regulation. The loss of SMARCB1 leads to genomic instability and impaired DNA repair, contributing to the aggressive nature and poor prognosis of these tumors [2]. Based on the anatomical location, MRTs are categorized into three subtypes: malignant rhabdoid tumor of the kidney (MRTK), atypical teratoid/rhabdoid tumor (AT/RT) in the central nervous system, and extrarenal extracranial rhabdoid tumor (EERT). Among these, EERTs are exceedingly rare, accounting for less than 5% of all pediatric soft tissue sarcomas [3].

EERTs typically manifest between 11 and 18 months of age and commonly arise in the chest wall, retroperitoneum, and liver. These tumors are characterized by their rapid progression, resistance to conventional therapies, and dismal prognosis, with a reported three-year survival rate of 23.71%. Most patients succumb to rapid tumor progression or recurrence within 8 months after diagnosis [4,5]. Histopathologically, EERTs have specific features, including filamentous cytoplasm, prominent nucleoli, and immunoreactivity for epithelial and mesenchymal markers such as vimentin, cytokeratin, and epithelial membrane antigen (EMA) [6].

Intrathoracic rhabdoid tumors, involving the mediastinum or pleura, are a rare subset of extracranial malignant rhabdoid tumors (EERTs), with an annual incidence of 0.6 per million children. The incidence is highest in the first year of life (5 per million) and decreases with age, reaching 0.04 per million in children aged 10 to 14 [7]. Only approximately 40 cases have been reported in the medical literature since their first description [8]. Their unique anatomical location presents distinct diagnostic and therapeutic challenges. These tumors typically manifest with respiratory symptoms such as dyspnea, cough, or pleural effusion, caused by rapid growth and mass effect within the thoracic cavity. Images usually show a large, heterogeneously enhancing intrathoracic mass; the radiological features often overlap with other pediatric thoracic malignancies, including germ cell tumors and sarcomas. This overlap frequently delays definitive diagnosis and treatment initiation, further affecting patient outcomes [6].

The management of intrathoracic rhabdoid tumors remains particularly challenging due to their anatomical location, aggressive behavior, and lack of standardized treatment protocols. Despite advances in multimodal therapy combining surgery, chemotherapy, and radiation, outcomes remain poor, with survival often measured in months even with aggressive intervention [5].

This case report presents several unique aspects that contribute significantly to the existing literature. First, our patient's presentation at six months of age is notably earlier than the typical age range reported for EERTs, potentially offering insights into early disease recognition. Second, the initial misdiagnosis as a germ cell tumor highlights a critical diagnostic challenge that has not been well documented in previous literature. Finally, the rapid progression to liver metastases despite multimodal therapy underscores the aggressive nature of these tumors and the urgent need for more effective treatment strategies.

By documenting the diagnostic challenges, treatment response, and disease progression in this case, we hope to deepen the understanding of intrathoracic rhabdoid tumors. This knowledge can help pediatric oncologists, radiologists, and pathologists recognize this rare but devastating disease earlier, potentially improving outcomes for future patients.

## Case presentation

A six-month-old male infant, born full-term via normal vaginal delivery to non-consanguineous parents with no significant family medical conditions reported, was brought to the ER with a three-day history of fever, persistent non-productive cough, and decreased oral intake. The parents reported progressive lethargy and increased work of breathing over the preceding 24 hours prior to admission. The infant had been previously healthy, with an uneventful prenatal and perinatal history and age-appropriate developmental milestones prior to the onset of symptoms.

On clinical examination, the infant appeared acutely ill and in respiratory distress, evidenced by tachypnea, intercostal retractions, and nasal flaring. Vital signs included a temperature of 38.5°C, respiratory rate of 60 breaths per minute, heart rate of 150 beats per minute, and oxygen saturation of 88% on room air. Chest auscultation revealed markedly diminished breath sounds over the left hemithorax and scattered crackles on the right. The cardiovascular examination was unremarkable, with no murmurs or abnormal heart sounds. The abdominal examination was normal, with no organomegaly or tenderness. Neurological assessment showed an alert but irritable infant without focal neurological deficits. Peripheral examination revealed no cyanosis, edema, or cutaneous abnormalities.

**Figure 1:**
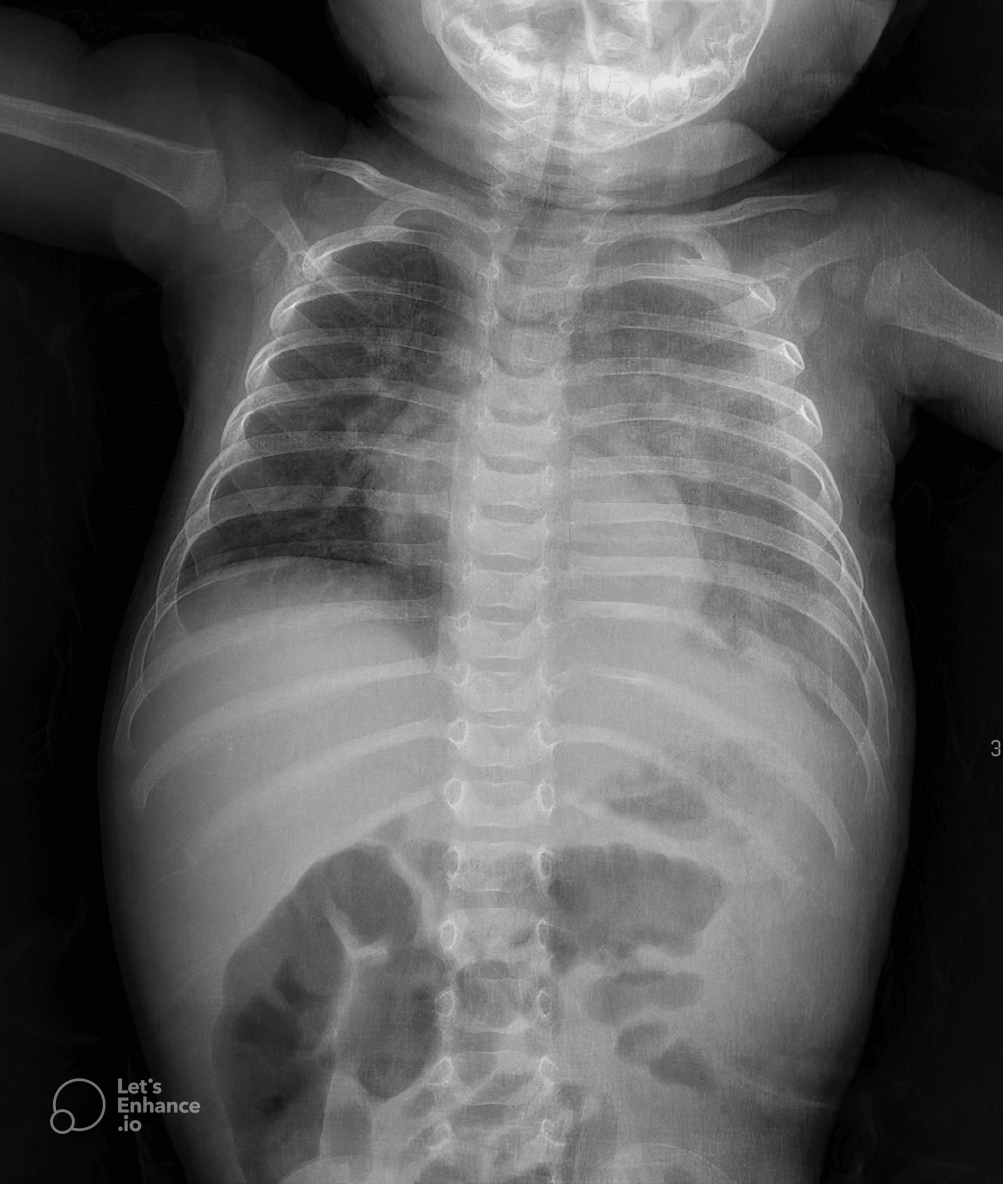
A large, homogeneous mass is observed occupying the left hemithorax, leading to significant mediastinal shift and compression of the right lung.

The clinical presentation raised concerns for a severe respiratory tract infection, prompting admission to the pediatric intensive care unit (PICU) for respiratory support with high-flow nasal cannula oxygen therapy. IV antibiotics and corticosteroids were initiated. A chest radiograph revealed a large, homogeneous mass occupying the left hemithorax, associated with mediastinal shift and compression of the right lung (Figure 1).

Laboratory investigations on admission (Table 1) revealed a normal WBC count and absolute neutrophil count, thrombocytosis, mild anemia, and elevated inflammatory markers. Renal and liver function tests were within normal limits, except for mild hyponatremia. Serum lactate dehydrogenase (LDH) was slightly elevated, while alpha fetoprotein (AFP) and beta HCG were within the normal range. Venous blood gases indicated compensated respiratory acidosis.

**Table 1:** Laboratory investigations on admission and reference ranges.

Lab Parameter	Patient's Value	Reference Range
WBC Count	13.3 × 10⁹/L	6-17.5 × 10⁹/L
Absolute Neutrophil Count (ANC)	8.1 × 10⁹/L	1.5-8.5 × 10⁹/L
Platelets	1154 × 10⁹/L	150-450 × 10⁹/L
Hemoglobin (Hb)	8.8 g/dL	10.5-12.8 g/dL
C-Reactive Protein (CRP)	8.7 mg/L	<5 mg/L
Erythrocyte Sedimentation Rate (ESR)	63 mm/h	<20 mm/h
Serum Creatinine	0.13 mg/dL	0.2-0.4 mg/dL
Alanine Aminotransferase (ALT)	5.1 U/L	<45 U/L
Aspartate Aminotransferase (AST)	17 U/L	<55 U/L
Total Bilirubin	0.13 mg/dL	<1.2 mg/dL
Sodium (Na)	128 mmol/L	135-145 mmol/L
Lactate Dehydrogenase (LDH)	557 U/L	<500 U/L
Alpha Fetoprotein (AFP)	11.5 ng/mL	0.6-12 ng/mL
Beta Human Chorionic Gonadotropin (β-HCG)	0.100 mIU/mL	<1.0 mIU/mL
pH	7.22	7.35-7.45
pCO₂	53.9 mmHg	35-48 mmHg
HCO₃	19.3 mmol/L	20-28 mmol/L

**Figure 2:**
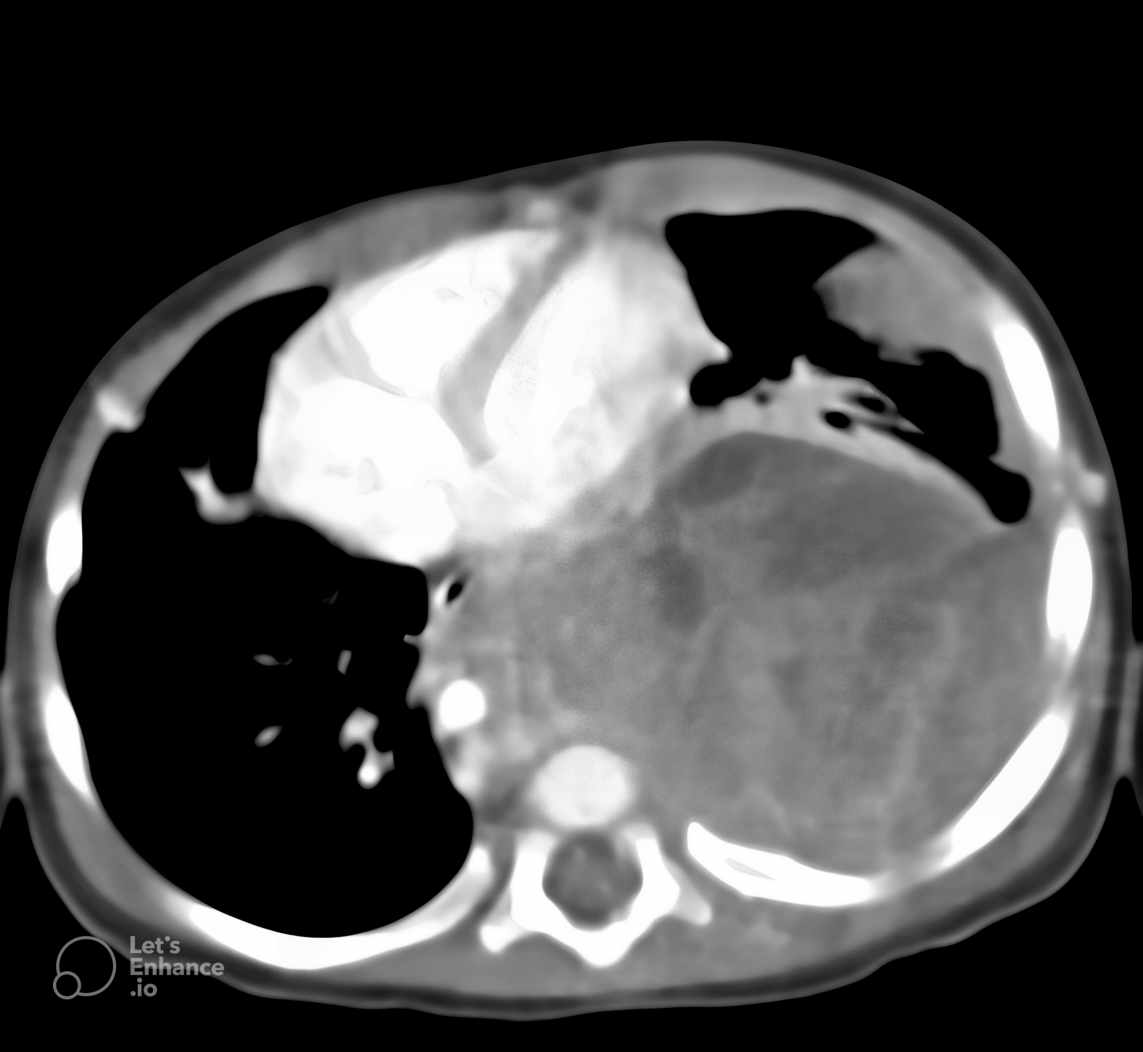
The initial CT scan of the chest reveals a well-circumscribed, heterogeneously enhancing soft tissue mass. The presence of heterogeneous enhancement suggests underlying necrosis or varying tissue composition, necessitating further histopathological correlation.

The infant’s respiratory condition deteriorated, necessitating intubation and mechanical ventilation. Subsequent CT of the chest identified a well-circumscribed, heterogeneously enhancing soft tissue mass, measuring approximately 110 × 77 × 60 mm, occupying the left thoracic cavity (Figure 2). The mass was pleural-based and extended into the pleural space, causing significant displacement of the heart and compression of the adjacent lung parenchyma.

The imaging findings raised concerns about an aggressive pleural tumor, such as a bronchopulmonary blastoma. Given the urgency, chemotherapy with the Vincristine, Actinomycin D and Cyclophosphamide (VAC) protocol, one cycle of Vincristine 0.05 mg/kg, Actinomycin 0.025 mg/kg, and Cyclophosphamide 50 mg/kg, was given. Over six days, the infant’s respiratory condition stabilized, and he was successfully extubated. This improvement allowed for a biopsy, which initially suggested a yolk sac germ cell tumor based on immunohistochemistry findings (positive for SALL-4, cytokeratin (CK AE1/AE3), and vimentin).

**Figure 3:**
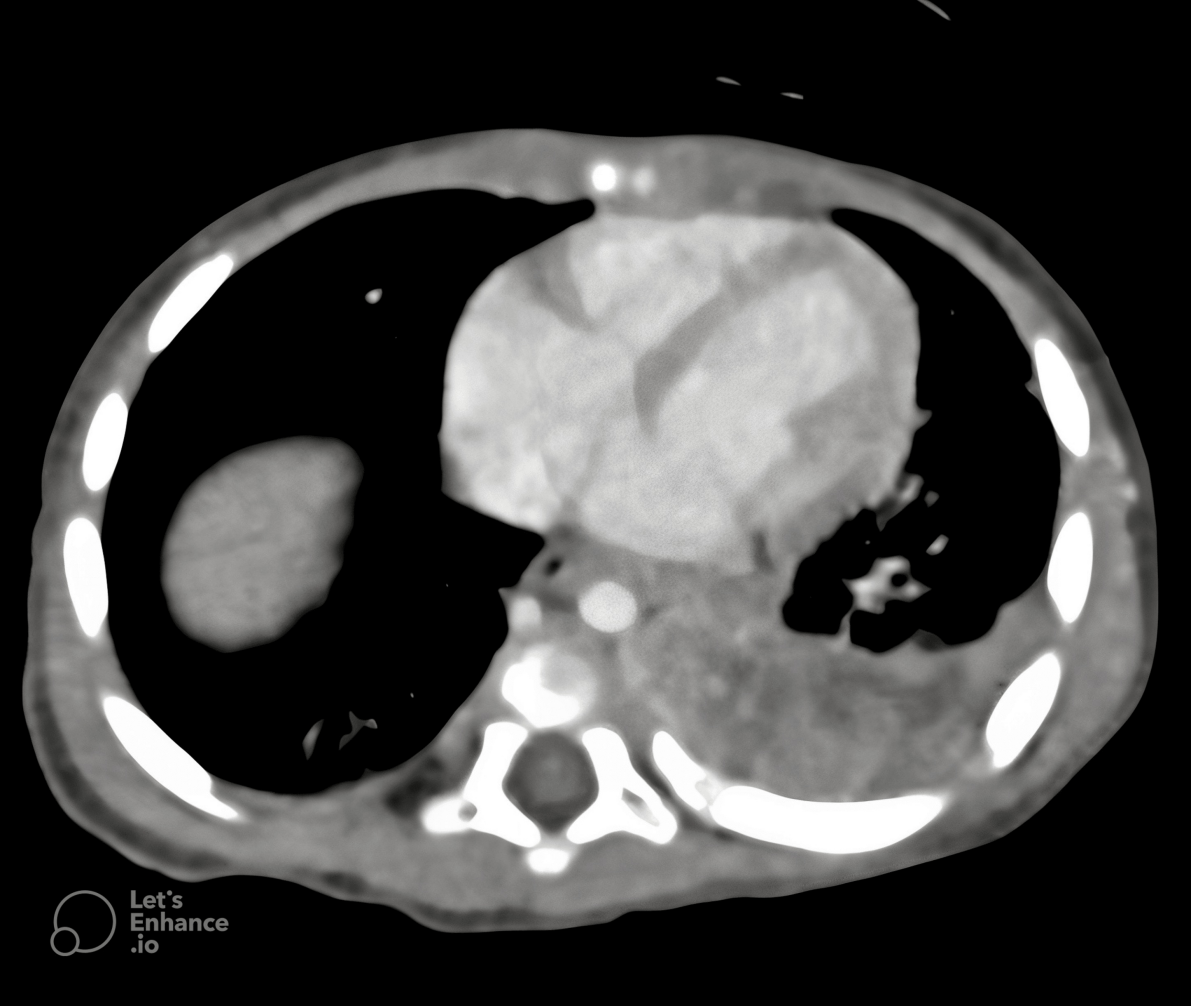
A follow-up chest CT, performed 14 days after initiating VAC chemotherapy, demonstrates a remarkable reduction in tumor size. VAC: Vincristine, Actinomycin D and Cyclophosphamide.

Follow-up CT after 14 days of VAC therapy showed a notable reduction in the tumor’s size to 80 × 55 × 50 mm (Figure 3). Based on this presumed diagnosis, treatment was transitioned to the BEP protocol: Bleomycin 15 units/m2, Etoposide 100 mg/m2, and Cisplatin 20 mg/m2. However, after two cycles of BEP, the tumor showed only minor additional shrinkage (to 70 × 50 × 50 mm), an atypical response for a germ cell tumor.

**Figure 4:**
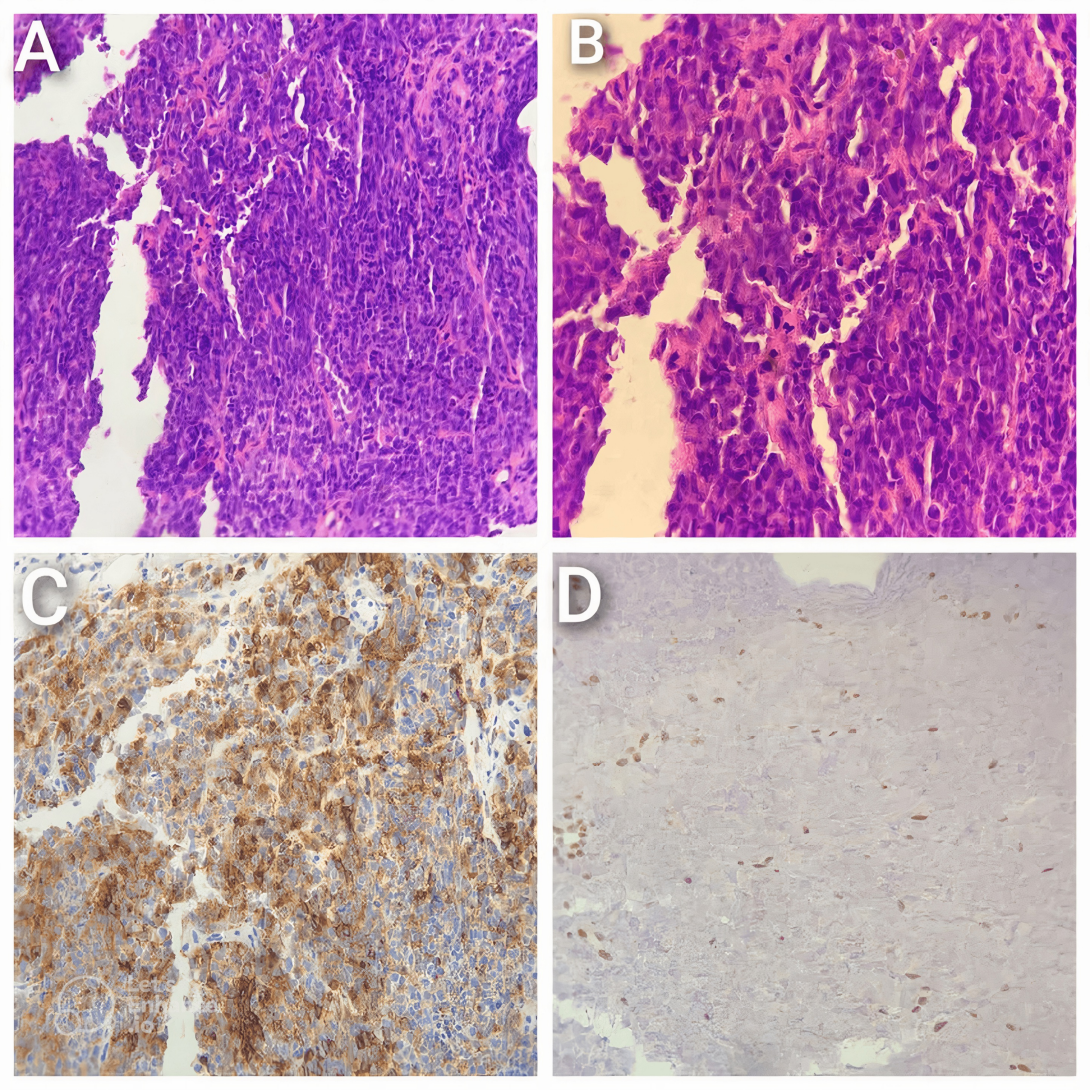
A: Low-power microscopic examination reveals sheets of polygonal cells with abundant glassy cytoplasm and prominent nucleoli, characteristic of the tumor’s histological architecture. B: High-power magnification highlights rhabdoid cells arranged in sheets, exhibiting eccentric round nuclei with distinct prominent nucleoli and frequent mitotic figures, indicative of an aggressive proliferative index. C: Immunohistochemical analysis shows EMA positivity in tumor cells, supporting epithelial differentiation. D: Nuclear SALL4 positivity in tumor cells further confirms the diagnosis, suggesting a germ cell or embryonal tumor origin. EMA: Epithelial Membrane Antigen; SALL4: Sal-like protein 4.

In light of the poor response to BEP, a second biopsy was performed. This time, histopathological analysis revealed poorly differentiated cells with rhabdoid features. IHC studies confirmed the diagnosis of MRT, characterized by the loss of SMARCB1/INI1 expression, a hallmark of MRT (Figure 4).

**Figure 5:**
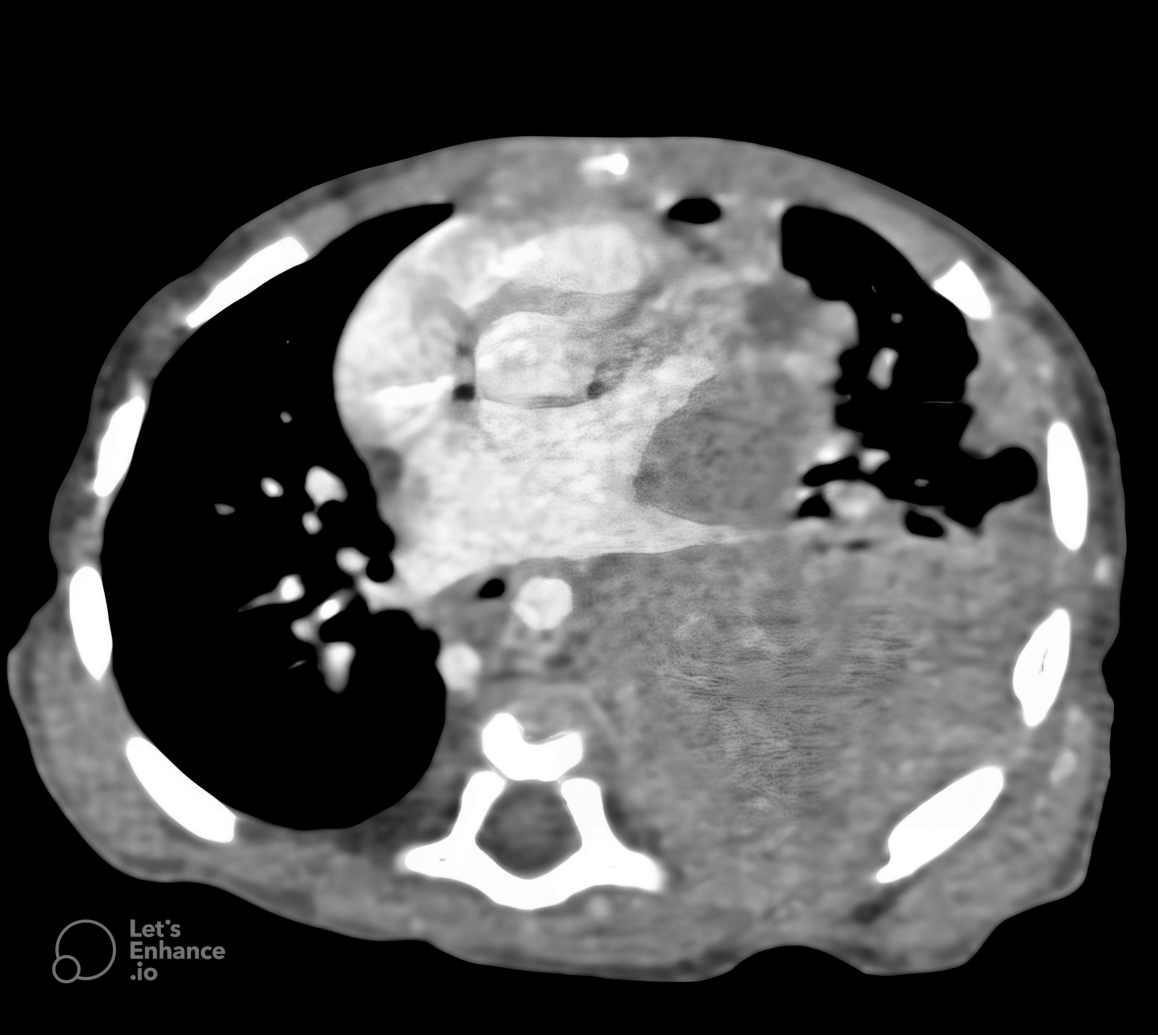
A subsequent chest CT illustrates progressive disease despite intensive chemotherapy, showing a noticeable increase in tumor size. This finding reflects the tumor’s resistance to conventional therapy, necessitating a reassessment of the treatment strategy.

With the diagnosis of MRT established, the treatment protocol was shifted to the European Rhabdoid Protocol [9]. Despite this aggressive treatment, follow-up imaging showed tumor progression, with the mass growing back to 110 × 70 × 60 mm (Figure 5).

Five months after the initial diagnosis, the child’s condition worsened significantly. His abdomen became distended, and splenomegaly was detected. Ultrasound imaging of the abdomen revealed two liver lesions, the largest measuring 15 × 11 mm, consistent with metastases. The tumor also compressed nearby organs, including the spleen and left kidney, but there was no evidence of direct invasion.

Despite aggressive multidisciplinary therapy, including chemotherapy and supportive care, the tumor exhibited refractory behavior. Surgery was not an option due to the tumor’s size and location. At 11 months of age, the infant’s condition has led to the transition to palliative care, focusing on his comfort and quality of life.

## Discussion

MRTs are rare and aggressive pediatric neoplasms that predominantly affect infants and young children. While most commonly found in the kidneys or central nervous system, EERTs can develop in other locations, including the thoracic cavity. Thoracic involvement accounts for approximately 12% of EERTs and is associated with particularly poor outcomes, with a 5-year survival rate of less than 15% [10]. Factors such as advanced stage at diagnosis and age under 1 year are significant predictors of a worse prognosis [4].

The rarity of intrathoracic MRTs makes timely diagnosis difficult and limits the establishment of standardized treatment protocols. For example, a previously reported case of an 18-month-old infant with a MRT of the chest wall highlighted the challenges of management, as the patient’s condition deteriorated rapidly despite the initiation of chemotherapy, culminating in death within two months of diagnosis [8].

In this case, diagnostic challenges were amplified by nonspecific presenting symptoms, including severe respiratory distress and poor feeding, which could mimic other pediatric conditions. Initial biopsy findings suggested a germ cell tumor based on immunoreactivity for markers such as SALL-4 and cytokeratin. However, the poor therapeutic response prompted a reevaluation, leading to a revised diagnosis of MRT based on rhabdoid morphology and loss of SMARCB1/INI1 expression [11]. This case highlights the critical role of molecular testing in distinguishing MRTs from other tumors with overlapping features. Early molecular characterization is vital to avoid delays in appropriate treatment and to minimize the exposure to ineffective and potentially harmful therapies.

The therapeutic course in this case reflects the inherent resistance of MRTs to conventional treatments. While the initial use of VAC (vincristine, actinomycin-D, cyclophosphamide) chemotherapy led to temporary tumor shrinkage and allowed for extubation, demonstrating its utility in emergencies [12], subsequent administration of BEP (bleomycin, etoposide, cisplatin), based on a provisional diagnosis of a yolk sac tumor, proved ineffective [13]. Following the definitive MRT diagnosis, the IC regimen (ifosfamide, carboplatin) was initiated, but tumor progression persisted. The rapid tumor progression highlights the therapeutic challenges posed by SMARCB1-deficient tumors, as supported by evidence showing limited benefit of ifosfamide-based regimens in high-risk MRTs [14]. In the reported case, no surgical resection or radiation therapy was performed due to rapid tumor progression, and palliative care interventions were initiated to address symptom management and family support.

Despite aggressive multimodal therapy, including surgery, chemotherapy, and radiation, the prognosis for MRTs remains dismal. Reported 3-year and 5-year overall survival rates for extracranial MRTs are approximately 23.7% and 18.4%, respectively [4]. The loss of SMARCB1, a key tumor suppressor gene, not only defines MRTs but also drives their aggressive biology and resistance to therapy [15].

Emerging therapies offer hope for improved outcomes. One promising approach is the use of Enhancer of Zeste Homolog 2 (EZH2) inhibitors, such as Tazemetostat, which target the epigenetic dysregulation caused by SMARCB1 deficiency [10]. Specifically, these inhibitors block the enzymatic activity of EZH2, a methyltransferase responsible for histone H3K27 methylation, which is overactive in the absence of SMARCB1. These inhibitors help restore normal gene expression and potentially make tumors more sensitive to treatment [16]. While Tazemetostat is currently approved for SMARCB1-deficient epithelioid sarcoma, its application in rhabdoid tumors is under investigation [15]. Early clinical trials have shown that Tazemetostat is well-tolerated and exhibits anti-tumor activity in SMARCB1-deficient tumors, with preliminary results suggesting efficacy in treating rhabdoid tumors [17].

Immune checkpoint inhibitors are another area of exploration. SMARCB1 loss may increase tumor mutational burden and immunogenicity, suggesting potential responsiveness to immunotherapy [18]. While preclinical data are encouraging, clinical studies are needed to determine their effectiveness in MRTs.

The impact of MRTs extends beyond medical challenges to significant psychosocial burdens on patients and families. The intense treatments, coupled with the high likelihood of poor outcomes, necessitate comprehensive psychosocial support. Early integration of palliative care is essential, focusing on symptom management, quality of life, and guidance through complex decision-making processes [19].

Addressing the challenges of MRT diagnosis and treatment requires a multifaceted approach. Developing better diagnostic biomarkers could facilitate earlier identification and intervention, while advancing molecularly targeted therapies and immunotherapies may improve treatment outcomes [20]. International collaboration through tumor registries and multicenter research initiatives is essential for deepening our understanding of these rare tumors and optimizing care [11]. These efforts could open the way for better prognoses and more comprehensive support systems for affected families.

## Conclusions

This case of intrathoracic rhabdoid tumor highlights key diagnostic and therapeutic challenges. Our experience emphasizes the critical importance of early SMARCB1/INI1 testing in young infants with aggressive thoracic masses, regardless of initial immunohistochemistry results. We recommend mandatory molecular testing, shorter interval imaging, and early integration of palliative care. Future research should focus on developing specific diagnostic markers and investigating novel therapeutic combinations. The rapid progression to liver metastases despite multimodal therapy underscores the urgent need for innovative treatment strategies to improve outcomes in these aggressive pediatric malignancies.

## References

[1] Sparano A, Kreiger P, Kazahaya K (2009). Malignant rhabdoid tumor of the parapharyngeal space. Ear Nose Throat J.

[2] Kalimuthu SN, Chetty R (2016). Gene of the month: SMARCB1. J Clin Pathol.

[3] Kohashi K, Tanaka Y, Kishimoto H (2016). Reclassification of rhabdoid tumor and pediatric undifferentiated/unclassified sarcoma with complete loss of SMARCB1/INI1 protein expression: three subtypes of rhabdoid tumor according to their histological features. Mod Pathol.

[4] Cheng H, Yang S, Cai S (2019). Clinical and prognostic characteristics of 53 cases of extracranial malignant rhabdoid tumor in children. A single-institute experience from 2007 to 2017. Oncologist.

[5] Madigan CE, Armenian SH, Malogolowkin MH, Mascarenhas L (2007). Extracranial malignant rhabdoid tumors in childhood: the Childrens Hospital Los Angeles experience. Cancer.

[6] Abdullah A, Patel Y, Lewis TJ, Elsamaloty H, Strobel S (2010). Extrarenal malignant rhabdoid tumors: radiologic findings with histopathologic correlation. Cancer Imaging.

[7] Tsipou H, Roka K, Gavra M, Glentis S, Stefanaki K, Kattamis AJ (2020). Congenital extracranial extrarenal rhabdoid tumor: a rare clinicopathologic entity and diagnostic challenge. J Case Rep Images Oncol.

[8] Mohamed DA, Essaber H, Waiss AA, Diekouadio F, El Haddad S, Fekkar A, Lamalmi N (2020). Pleural effusion revealing a malignant rhabdoid tumor of the chest wall in an infant: a case report and literature review. Int J Case Rep Images.

[9] (2021). European Rhabdoid Registry. A multinational registry for rhabdoid tumors of any anatomical site. University Hospital Augsburg, Department of Pediatrics and Adolescent Medicine, Version 6, August 17. https://www.gpoh.de/kinderkrebsinfo/content/e1676/e9032/e1758/e83294/download84621/EU-RHABProtokoll_2021_08_ger.pdf.

[10] (2025). PathologyOutlines.com. (n.d.). Rhabdoid tumor. https://www.pathologyoutlines.com/topic/softtissuerhabdoidtumor.html.

[11] Roberts CW, Biegel JA (2009). The role of SMARCB1/INI1 in development of rhabdoid tumor. Cancer Biol Ther.

[12] (2025). Cancer Research UK. (n.d.). VAC chemotherapy. https://www.cancerresearchuk.org/about-cancer/treatment/drugs/vac.

[13] (2025). Cleveland Clinic. (n.d.). Yolk sac tumor. https://my.clevelandclinic.org/health/diseases/yolk-sac-tumor.

[14] Wong CI, Benedetti DJ, Kao PC, Ma C, Marcus KJ, Mullen EA (2023). Tolerability of ifosfamide-containing regimen in patients with high-risk renal and INI-1-deficient tumors. Pediatr Blood Cancer.

[15] Lanzi C, Arrighetti N, Pasquali S, Cassinelli G (2023). Targeting EZH2 in SMARCB1-deficient sarcomas: advances and opportunities to potentiate the efficacy of EZH2 inhibitors. Biochem Pharmacol.

[16] Feng S, Marhon SA, Sokolowski DJ (2024). Inhibiting EZH2 targets atypical teratoid rhabdoid tumor by triggering viral mimicry via both RNA and DNA sensing pathways. Nat Commun.

[17] Chi SN, Yi JS, Williams PM (2023). Tazemetostat for tumors harboring SMARCB1/SMARCA4 or EZH2 alterations: results from NCI-COG pediatric MATCH APEC1621C. J Natl Cancer Inst.

[18] Ngo C, Postel-Vinay S (2022). Immunotherapy for SMARCB1-deficient sarcomas: current evidence and future developments. Biomedicines.

[19] Lim SM, Kim HC, Lee S (2013). Psychosocial impact of cancer patients on their family members. Cancer Res Treat.

[20] Alva E, Rubens J, Chi S, Rosenberg T, Reddy A, Raabe EH, Margol A (2023). Recent progress and novel approaches to treating atypical teratoid rhabdoid tumor. Neoplasia.

